# The Structure, Vibrational Spectra, and Thermal Expansion Study of AVO_4_ (A=Bi, Fe, Cr) and Co_2_V_2_O_7_

**DOI:** 10.3390/ma13071628

**Published:** 2020-04-01

**Authors:** Xiaoke He, Chenjun Zhang, Ding Tian

**Affiliations:** 1 School of Electric Power, North China University of Water Resources and Electric Power, 450045, China; 18848968152@163.com; 2 National Research Center of Pumps, Jiangsu University, Zhenjiang, 212013, China; zcj5503@163.com; 3 School of Mechanical Engineering, Luoyang Institute of Science and Technology, Luoyang, 471023, China

**Keywords:** vanadium, Raman spectroscopy, thermal expansion, crystal structure

## Abstract

Vanadate is an important functional material. It has been widely studied and applied in luminescence and photocatalysis. Vanadium compounds have been synthesized to investigate the thermal expansion properties and structure. Both BiVO_4_ and Co_2_V_2_O_7_ are monoclinic at room temperature, FeVO_4_’s crystal structure is triclinic, and CrVO_4_ is orthorhombic. The relatively linear, thermal-expansion, and temperature-dependent Raman spectroscopy results showed that the phase transition of BiVO_4_ occurred at 200 to 300 °C. The coefficient of thermal expansion (CTE) of Co_2_V_2_O_7_ was larger than that of the monoclinic structure BiVO_4_. The CTE of the tetragonal structure of BiVO_4_ was 15.27 × 10^−6^ °C^−1^ which was the largest CTE in our measurement results, and the CTE of anorthic structure FeVO_4_ was 2.84 × 10^−6^ °C^−1^ and was the smallest.

## 1. Introduction

Due to the multivalent of vanadium, vanadate has rich physical and chemical properties. Vanadate is a kind of important functional material; it has been widely studied and applied in luminescence and photocatalysis. The bandgap of BiVO_4_ is approximately 2.0 eV which means that it is a classic semiconductor. Bidmuth vanadate (BiVO_4_) is a polycrystalline compound, among which there are three kinds of crystal structures: monoclinic, orthorhombic, and tetragonal structure. The tetragonal structure has absorption band in the ultraviolet region, while the monoclinic structure has absorption band in the visible region as well as in the ultraviolet region. Vanadate (BiVO_4_) has emerged as a very photoanode for photoelectrochemical water splitting [1,2,3,4,5]. Although the hole/electron pair, produced by the excitation of BiVO_4_, has strong redox ability; it also has some disadvantages for practical application: a high electron hole recombination rate, low photocatalytic efficiency, small particles which are easy to lose and difficult to recover, etc. As an n-type semiconductor material, the bandgap of FeVO_4_ is approximately 1.9–2.7 eV. There are four crystal types of FeVO_4_, only triclinic structure material is easy to obtain [6]. The triclinic structure of FeVO_4_ remains up to approximately 3 GPa, and then a first-order phase transition to a new monoclinic with space group C2/m is observed [7]. As a kind of transition metal oxide, FeVO_4_ can be used as electrode materials for ion batteries and supercapacitors [8,9,10,11]. The compound materials of FeVO_4_/BiVO_4_ and FeVO_4_/V_2_O_5_ has higher than pure FeVO_4_ photocatalytic activity [12,13]. Chrome vanadate has three different crystal forms tetragonal, monoclinic, and orthorhombic structures [7]. The ambient-pressure stable polymorph of CrVO_4_ is orthorhombic space group $D_{2h}^{17}$-Cmcm-, with Z=4 at room temperature [14]. Cobalt vanadates and their composites have drawn a tremendous amount of attention because of their outstanding cycling stability [15,16]. The Co_2_V_2_O_7_ was recently reported to exhibit amazing magnetic field-induced magnetization plateaus and ferroelectricity, but its magnetic ground state remains ambiguous due to the fact of its structural complexity [17]. 

From the above discussion, we know that vanadium compounds have many structures, rich physical and chemical properties which makes them have potential application value in many aspects. Although there are many studies on the structure and application of vanadium compounds, the thermal expansion of materials has not been reported very intensively. It has been pointed out that ionic radius and electronegativity of the cations are important with respect to structure and phase transition temperature [18,19]. Herein, we have prepared some vanadate materials by a simple solid-phase sintering method, X-ray diffraction (XRD) was used to measure the structure of materials, Raman scattering was used to measure the lattice vibration, and dilatometers was used to measure the thermal expansion.

## 2. Experimental and Methods

The AVO_4_ and Co_2_V_2_O_7_ were synthesized by a solid-state method from Fe_2_O_3_ (≥99.0%), Bi_2_O_3_ (≥99.0%), Cr_2_O_3_ (≥99.0%), Co_2_O_3_ (≥99.5%), and V_2_O_5_ (≥99.0%). The raw materials were mixed according to stoichiometric amounts (1:1) of A_2_O_3_ and 2%–5% excess V_2_O_5_ of desirable material except (in order to compensate for the loss in the sintering process) and ground in a mortar for 2 h. Then, alcohol was poured over the raw material and grind again until dry. Lastly, it was pressed into tablets with a length of approximately 7 mm and a diameter of approximately 6 mm, followed by sintering at 750 °C for 4 h and cooling naturally to room temperature.

The XRD measurements were carried out with an X’Pert PRO X-ray Diffractometer (Bruker D8, Bruker, Karlsruhe, Germany). Raman spectroscopy (Renishaw MR-2000 Raman spectrometer, Gloucestershire, UK) with a TMS 94 heating/freezing stage with an accuracy of ±0.1 °C was used to characterize the vibrational property of lattice. The linear thermal expansion coefficients were measured on dilatometers (LINSEIS DIL L76, Linseis, Selb, Germany), with heating and cooling rates of 5 °C/min.

## 3. Results and Discussion

### 3.1. Crystal Structure Analysis

**Figure 1:**
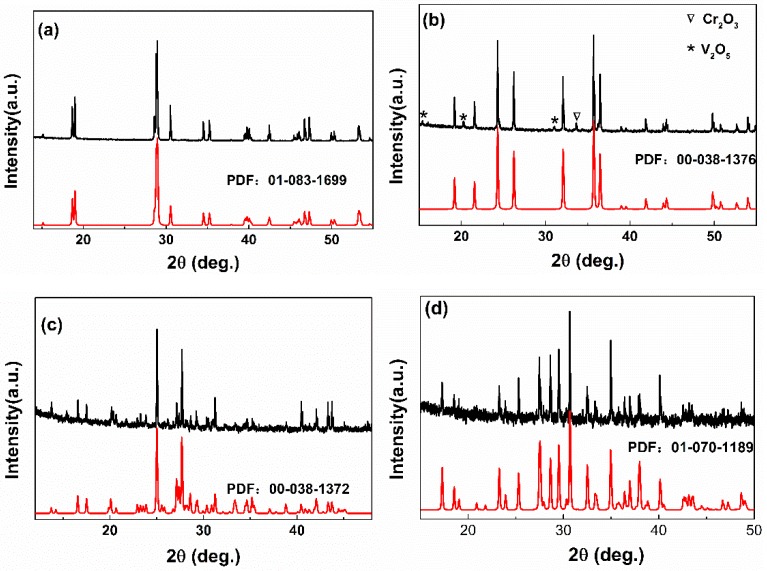
X-ray diffraction pattern of (**a**) BiVO_4_, (**b**) CrVO_4_, (**c**) FeVO_4_, and (**d**) Co_2_V_2_O_7._

Figure 1 shows the XRD patterns of as-prepared materials. Figure 1a is the pattern of BiVO_4_, all diffraction peaks corresponded to BiVO_4_ (PDF No. 01-083-1699) which means that the material crystals were in monoclinic structure space group I2/b, with Z = 4. The lattice constants of BiVO_4_ were a = 5.196 Å, b = 5.094 Å, c = 11.703 Å and α = β = 90°, γ = 90.380°. Figure 1b is the pattern of CrVO_4_, the primary diffraction peaks corresponded to CrVO_4_ (PDF No. 00-038-1376, space group Amam) except for weak peaks indicated as “∇” for secondary phase Cr_2_O_3_ and “∗” for secondary phase V_2_O_5_ which could relate to the fact that the reaction time was much shorter than that reported in the literature (122 h). There was a second and third phase which could lead to some situations, such as internal stress, many cracks on the tablet, etc. The material crystals in orthorhombic structure with space group Amam from the primary diffraction peaks. The lattice constants of CrVO_4_ are a = 5.567 Å, b = 8.210 Å, c = 5.975 Å. The pattern of FeVO_4_ is shown in Figure 1c. As seen, the diffraction peaks are corresponding to FeVO_4_ (PDF No. 00-038-1372, space group P-1) which crystal in anorthic structure. The lattice constants of FeVO_4_ are a = 6.720 Å, b = 8.059 Å, c = 9.256 Å, and α = 96.7°, β = 106.4°, γ = 101.6°. Figure 1d is the pattern of Co_2_V_2_O_7_, all diffraction peaks corresponded to Co_2_V_2_O_7_ (PDF No. 01-070-1189) with lattice constants a = 6.595 Å, b = 8.380 Å, and c = 9.470 Å which means that the material crystals were in monoclinic structure space group P21/c, with Z = 4.

**Figure 2:**
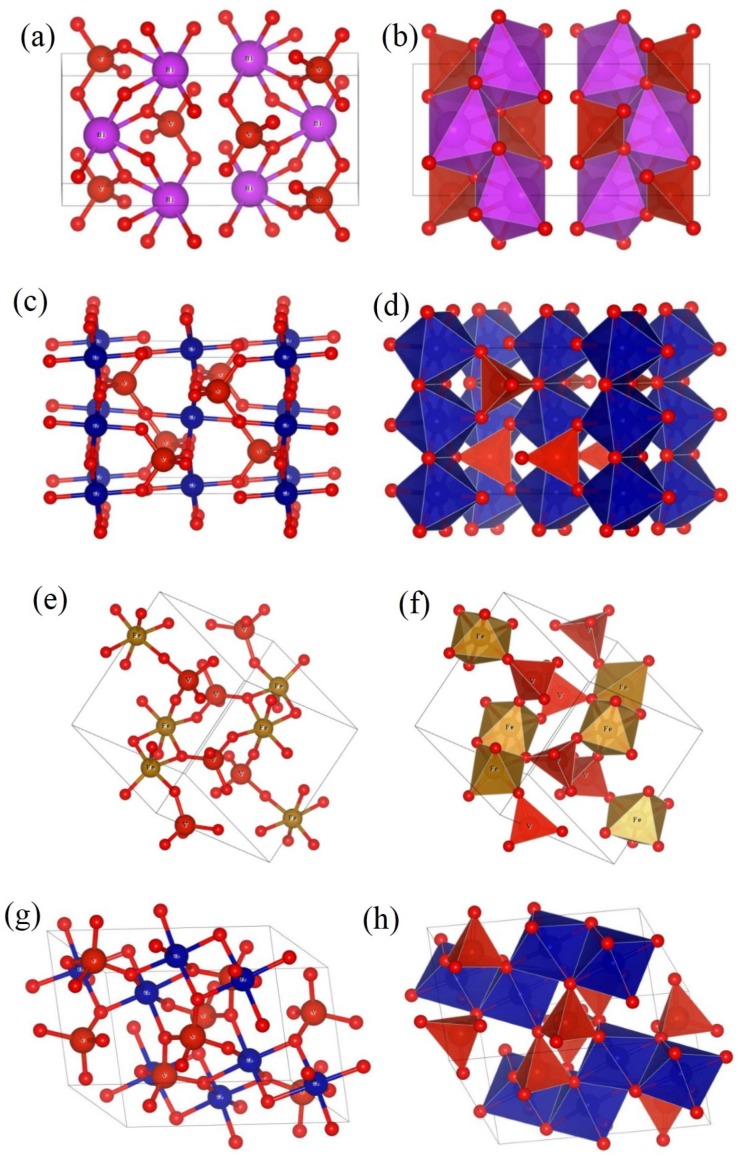
Crystal structure of (**a**,**b**) BiVO_4_, (**c**,**d**) CrVO_4_, (**e**,**f**) FeVO_4_, and (**g**,**h**) Co_2_V_2_O_7_.

To visualize the coordination number associated with the structural transitions, crystal structures of monoclinic (BiVO_4_, Co_2_V_2_O_7_), triclinic (FeVO_4_), and orthorhombic (CrVO_4_) systems with polyhedral representation were drawn using a VESTA software as shown in Figure 2 (the “atomic coordinates” were obtained come from the joint conferences on pervasive computing (JCPC) references). The BiVO_4_ crystal structure was monoclinic. From Figure 2a,b, Bi and V atoms occupied the symmetry position 4e, and O atoms occupied 8f. The distance between the V and O atoms was evenly distributed (approximately 1.68 Å and 1.785 Å); however, the distance of the Bi and O was very variable. There are six symmetry VO_4_ tetrahedras and asymmetrical BiO_6_ octahedra in one primitive cell of BiVO_4_. The CrVO_4_ crystal structure was orthorhombic (Figure 2c,d). The distance between the V and O atoms (approximately 1.63342 Å and 1.70978 Å) was shorter than that of BiVO_4_, and the distances between the Cr and O atoms were 1.98422 Å and 2.04868 Å. It can be seen that there are four CrO_6_ octahedra around each tetrahedron; however, each CrO_6_ octahedron is not only connected with six tetrahedron vertices, but also connected with two other octahedron edges. The FeVO_4_ crystal structure was triclinic Figure 2d. For FeVO_4_, there were 18 symmetrical inequivalent atoms in a one-unit cell, and all the atoms occupied the symmetry position 2i (Figure 2e). The total number of atoms in a unit cell was 36. The distance between the V atom and the O atom was different. The unit cell contained three asymmetrical inequivalent VO_4_ tetrahedra, two asymmetrical inequivalent FeO_6_ octahedra, and one FeO_5_ polyhedron [20] (Figure 2f). The CoV_2_O_7_ crystal structure was monoclinic Figure 2g, the total number of atoms in a unit cell was 44. The distance between the V atom and the O atom was different. The unit cell contained three symmetrical VO_4_ of each tetrahedron and six asymmetrical CoO_6_ octahedra (Figure 2h). It can be seen that each VO_4_ tetrahedron was connected to four CoO_6_ octahedra by the O atom; however, each CrO_6_ octahedron was connected by the O atom to six VO_4_ tetrahedras and shared a common edge with two other octahedral.

### 3.2. Thermal Expansion Property

**Figure 3:**
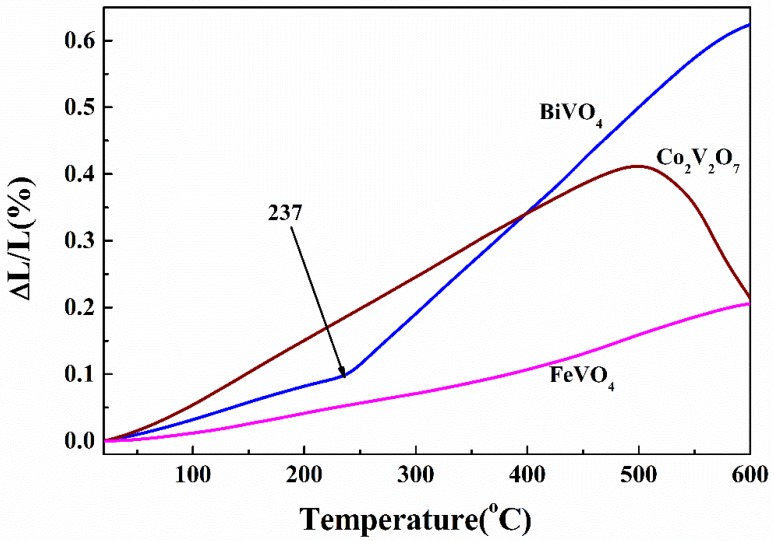
Relative length change of BiVO_4_, FeVO_4_, and Co_2_V_2_O_7._

Figure 3 shows the relative linear thermal expansion of BiVO_4_, FeVO_4_, and Co_2_V_2_O_7_. It was found that the samples have different relative linear thermal expansion. For BiVO_4_, there was a thermal expansion inflection point at about 237 °C which means that the material occurs phase transition at the temperature. The coefficients of thermal expansion (CTEs) were calculated as the average linear thermal expansion coefficient in terms of the slope of thermal expansion versus temperature. The CTEs were measured to be (4.664 ± 0.005) × 10^−6^ °C^−1^ (25–235 °C) and (15.40 ± 0.002) × 10^−6^ °C^−1^ (240–550 °C). For FeVO_4_, there was a gradual change thermal expansion at approximately 400 °C, the CTE of FeVO_4_ was obtained to be (2.751 ± 0.004) × 10^−6^ °C^−1^ from 20 to 350 °C and (5.245 ± 0.005) ×10^−6^ °C^−1^ from 400 to 600 °C. Although both vanadium and iron are variable metals and thermal expansion is related to valence states [21], we prepared and measured the material in an air atmosphere, vanadium and iron should remain stable in the highest valence state. So, there should be no chemical expansion here. The thermal expansion of Co_2_V_2_O_7_ was stable below 500 °C, and the CTE was (9.230 ± 0.004) × 10^−6^ °C^−1^ from 20 to 500 °C. The inflection point above 500 °C is due to the softening of glass state above 500 °C which can be explained by the fact that Co_2_V_2_O_7_ goes from the crystalline form to a glassy one. This phenomenon indicates that material intelligent stability exists with below 500 °C. Though the structure of Co_2_V_2_O_7_ is similar to BiVO_4_, their CTE is very different. This could come from the different ionic radius of Co^3+^ (63 pm) and Bi^3+^ (108 pm). The ionic radius of Co^3+^ (63 pm) equals that of Fe^3+^ (64 pm); however, they had the largest difference in CTE in this study. This was due to the different structures.

**Figure 4:**
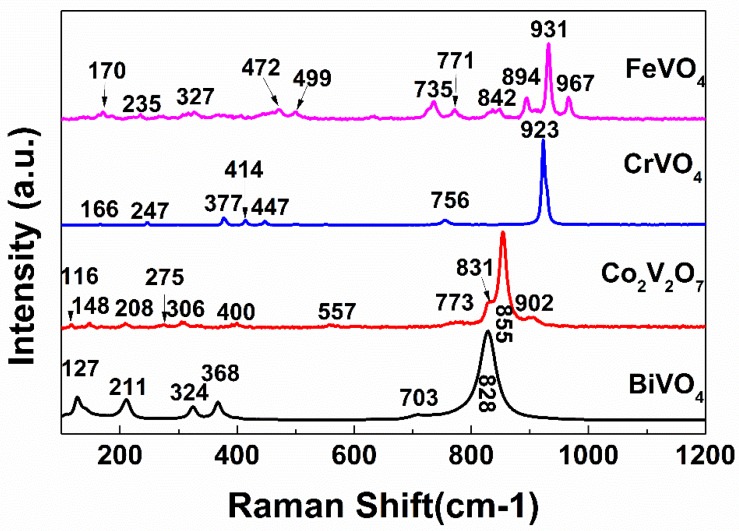
Raman spectra of BiVO_4_, FeVO_4_, CrVO_4_, and Co_2_V_2_O_7_.

Raman spectroscopy was applied to further demonstrate the existence of crystal. Raman spectra collected at room temperature is shown in Figure 4. The Raman spectra of BiVO_4_, CrVO_4_, FeVO_4_, and Co_2_V_2_O_7_ were in agreement with literature [10,20,22,23] and the spectra did not show the characteristic bands of V_2_O_5_. Hence, no effort was taken to consider the product selectivity in this work. For BiVO_4_, the primitive cell contained 28 atoms (Figure 2) and, in principle, 81 vibrational modes were expected. The band at approximately 828 cm^−1^ corresponded to stretching modes of V–O bonds, and there was no splitting which means that degeneracy occurs in the symmetric stretching vibration of VO_4_ tetrahedron. The strongest peak of CrVO_4_ and FeVO_4_ was much higher, whereas the stretching modes of V–O give rise to intense bands, the difference in electronegativity of these metal (Bi, Co, Cr, and Fe). The Raman bands of FeVO_4_ were much more than that of BiVO_4_, CrVO_4_ and Co_2_V_2_O_7_, because the structure of FeVO_4_ is triclinic, and all vibrations are nondegenerate. The 36 atoms in the unit cell had 105 vibrational modes among which 54 optical modes were Raman active A*_g_* modes, 51 were infrared active A*_u_* modes [23]. For all Raman spectra, the stretching modes of V–O combining M–O and V–O occurred above 650 cm^−1^, and bending modes together with stretching modes appeared in the 630–420 cm^−1^ region. The lower wavenumber bands were external modes from lattice, translational, and vibrational motions [24,25]. From a crystalline perspective, all catalysts were composed of V–O polyhedrons and other metal–oxygen polyhedrons.

**Figure 5:**
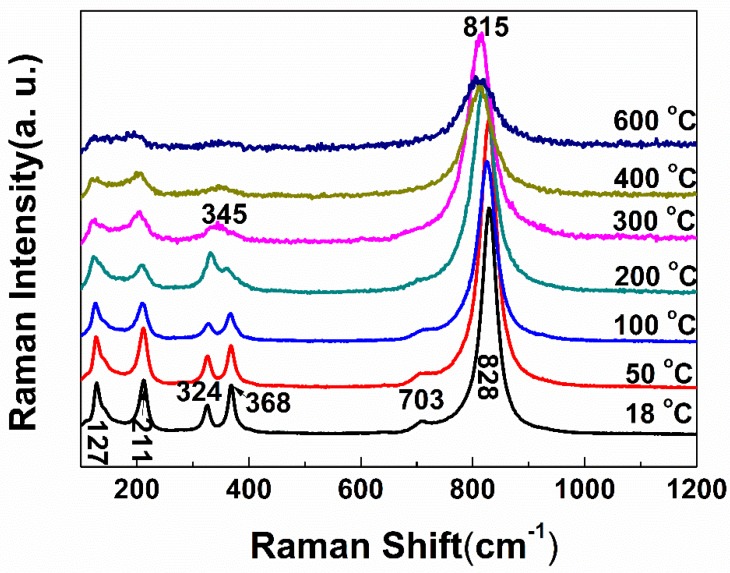
Raman spectra of BiVO_4_ at temperature of 18, 50, 100, 200, 300, 400, and 600 °C.

In order to study the sudden change in thermal expansion of BiVO_4_, the Raman spectroscopy dependent temperature of BiVO_4_ is shown in Figure 5. The Raman bands became weaker and weaker with the increasing temperature which reflects the increase of the degree of disordering of the crystal structure. The relative intensity of 368 and 324 cm^−1^ had obvious change at 200 °C, and they disappeared at 300 °C; meanwhile, there was a new band at approximately 345 cm^−1^. There was not only one change. The bands at 127 and 211 cm^−1^ gradually became a wave packet; meanwhile, the 703 cm^−1^ band disappeared, and the 828 cm^−1^ band moved to 815 cm^−1^, this might be caused by the bond expansion and weakening. All these mean that there was a phase transition between 200 °C and 300 °C. There was no change in the Raman spectra above 300 °C. The high temperature Raman spectra were in agreement with tetragonal structure which means that BiVO_4_ crystal tetragonal as well [26]. Compared with Figure 3, we found that materials with high symmetry have larger CTE. The Raman band at 368 and 324 cm^−1^ could inhibit the thermal expansion of material. It means that the thermal expansion property was related to the structure of the material.

## 4. Summary

Vanadium compounds were synthesized to investigate the thermal expansion properties and structure. The CTE of Co_2_V_2_O_7_ was bigger than monoclinic structure BiVO_4_ which means that the thermal expansion property was related to the ionic radius of metals. The CTE of the tetragonal structure of BiVO_4_ was 15.27 × 10^−6^ °C^−1^ which was the biggest CTE in our measurement results, and the CTE of tetragonal structure FeVO_4_ was 2.84 × 10^−6^ °C^−1^ which was the smallest. This indicates that the thermal expansion property was related to the structure of the material.
